# Inhibitory effects of taraxasterol and aqueous extract of *Taraxacum officinale* on calcium oxalate crystallization: *in vitro* study

**DOI:** 10.1080/0886022X.2018.1455595

**Published:** 2018-04-05

**Authors:** Mahboubeh Yousefi Ghale-Salimi, Maryam Eidi, Nasser Ghaemi, Ramezan Ali Khavari-Nejad

**Affiliations:** a Department of Biology, College of Basic Sciences, Science and Research Branch, Islamic Azad University, Tehran, Iran;; b Department of Biology, College of Biological Sciences, Varamin-Pishva Branch, Islamic Azad University, Varamin-Pishva, Iran;; c School of Chemistry, College of Science, University of Tehran, Tehran, Iran

**Keywords:** Dandelion, taraxasterol, kidney stone, calcium oxalate, crystal, urine

## Abstract

**Objective:** We investigated and compared the effects of taraxasterol, aqueous extract of *T. officinale* (AET) aerial part, and potassium citrate (PC) on calcium oxalate (CaOx) crystallization *in vitro*.

**Materials and methods:** CaOx crystallization was induced by adding sodium oxalate to synthetic urine. Taraxasterol (2.5, 5, 7.5 and 12.5 μg/mL), extract (1, 2, 4 and 8 mg/mL), and PC (100, 150, 200 and 350 mg/mL) were subjected to anti-crystallization activities. The absorbance and %inhibition of nucleation of CaOx crystals were evaluated by spectrophotometer at 2, 4, 6, 8, 10, 20, 30, 40, 50 and 60 min and the number and morphology of crystals were studied by light microscopy after 60 min.

**Results:** Presence of taraxasterol, extract and PC decreased absorbance in experimental samples compared to control, significantly. The nucleation of crystals is inhibited by taraxasterol, extract, and PC (26–64, 55–63 and 60–70%, respectively). The number of CaOx crystals were decreased in presence of taraxasterol (*p* < .01), extract (*p* < .001), and PC (*p* < .001) in a dose-dependent manner. Presence of taraxasterol, extract, and PC decreased the number of CaC_2_O_4_ monohydrate, while increased CaC_2_O_4_ dihydrate crystals, significantly. Also, the diameter of CaC_2_O_4_ dihydrate crystals was decreased in presence of taraxasterol, extract and PC, significantly.

**Conclusions:** This research indicated that taraxasterol and extract have anti-crystallization activities and effectiveness of the extract is more potent than taraxasterol. It could be because of another constituent in the extract with the synergistic effect.

## Introduction

The worldwide incidence of urolithiasis is increasing and calcium oxalate (CaOx) is the primary constituent of the majority of stones formed in the urinary system of patients with urolithiasis. Urolithiasis is the formation of stones in the urinary tract that causes variable degrees of pain, bleeding, and leads to secondary infection [1].

Significant development has been achieved in the management of urolithiasis following the introduction of several modern techniques, including extracorporeal lithotripsy, and nephrolithotomy. However, stone fragments that are retained after treatment may serve as nidi for the formation of new kidney stones. Therefore, effective therapies to prevent recurrence of the disease are still required. Unfortunately, despite considerable progress in medical therapy, there is no satisfactory drug or therapy to treat kidney stones [2].

It is reported the prevalence rate varies from 2% to 13% in developed countries to 0.5–1% in developing countries [1]. Areas with the higher incidence of kidney stones are Scandinavian countries, Mediterranean countries, British Islets, Northern Australia, Central Europe, portions of the Malayan Peninsula, China, Pakistan, and Southern Iran [1,3]. Iran has a high incidence of urinary calculi, particularly in Southern areas [3]. CaOx urolithiasis accounts for approximately 75% of urinary stone disease in the United States [1]. Many studies have also indicated that CaOx stones are the major constituents of renal calculi [4–6]. There are two different kinds of CaOx_4_ stones, CaC_2_O_4_ monohydrate (COM) and CaC_2_O_4_ dihydrate (COD) crystals. COM is thermodynamically more stable than COD and usually observed in clinical cases and has more affinity for renal tubular cells than COD [4].

In fact, urolithiasis is a complicated process and result of multiple physicochemical events including supersaturation, nucleation, growth, aggregation, and retention of calculi in the renal tubules. The supersaturation of urine results in nucleation which is defined as the formation of the solid crystals in urine [7]. Crystal growth occurs after supersaturation and then primary nucleus of kidney stone was formed. Crystal aggregation is affected by viscous bindings, such as external crystalline compounds with multiple binding sites. Finally, aggregated crystals are retained in the urinary system and attached to epithelial cells of the renal tubules. Thus, one way to inhibit stone formation would be to prevent crystal retention, nucleation, and aggregation [8].

The surgical operation, lithotripsy, and local calculus disruption using high-power laser are widely used to remove the calculi. However, these procedures are highly expensive and recurrence of disease is quite common [9]. The recurrence rate without preventive treatment is ∼10% at 1 year, 33% at 5 years and 50% at 10 years [10].

Therefore, effective therapies to prevent stone recurrence are still required. Unfortunately, despite considerable progress in medical therapy, there is no satisfactory drug to treat kidney stones. Thus, it appears useful to search for new therapy which is used either alone or in combination with usual existing methods.

In this regard, traditional herbal medicine can be a potent source of new anti-urolithiatic remedies, because it is shown that their extracts and compounds have biological activities. A number of plants have been used which claim to treat the kidney stone [4]. It is reported the plants promote the nucleation of CaC_2_O_4_ crystals, increase their number but decrease their size [11].

*Taraxacum officinale* L. [dandelion (Compositae)] is commonly known as Ghasedak in the Iranian herbal medicine. *T. officinale* has long been used in traditional medicine for its lactating, choleretic, diuretic, anti-angiogenic, anti-rheumatic, and anti-inflammatory properties [12–15], but its anti-urolithiatic activity and significant constituents have not been studied, completely.

Taraxasterol, a pentacyclic-triterpene compound which is isolated from *T. officinale* [16], and its anti-urolithiatic effect is unknown. Since potassium citrate is known for preventing the formation of kidney stone [17], in this study the *in vitro* anti-crystallization activities of taraxasterol, aqueous extract of *T. officinale* aerial parts has been investigated and compared with potassium citrate.

## Materials and methods

### Chemicals

Taraxasterol was purchased from Chem Face Co, China. All the other chemicals used in the study were of analytical grade and procured from Merck, Germany.

### Preparation of extract

Dandelion aerial parts were collected from the Kashan area in the summer 2016 and were scientifically approved in the Department of Botany of Islamic Azad University (Voucher number: 047,643, deposited in I.A.U. Herbarium, identified by Prof. Ramezan Ali Khavari-Nejad). The dried aerial parts (300 g) were grounded (500 µm) and extracted three times (48 h) with distilled water (2500 mL), and filtered through a glass filter funnel. The extract was combined and the water was evaporated under reduced pressure by rotavapor, and the rest was mixed and homogenized [11,18].

### Determination of total phenolics

The amount of total phenolics in the extract was determined using the Folin–Ciocalteu colorimetric method, according to a described procedure [18].

### Preparation of Folin–Ciocalteu’s phenol reagent

Sodium tungstate (100 g) and 25 g of sodium molybdate were dissolved in 800 mL of water in a 1500 mL flask. Then, 50 mL of phosphoric acid and 100 mL of HCl were added and refluxed for 10 h. After cooling and adding 150 g of lithium sulfate, 50 mL of water and 4–6 drops of bromine water were added and allowed to stand for 2 h. Then, the solution was boiled for 15 min and cooled before filtration. The reagent should have no greenish tint [18].

### Determination of total phenolic contents

The amount of total phenolics in the extract was determined with the Folin–Ciocalteu reagent. Gallic acid was used as a standard and the total phenolics were expressed as mg/g gallic acid equivalents (GAE). Concentrations of 0.01, 0.02, 0.03, 0.04 and 0.05 mg/mL of gallic acid were prepared. Also, concentrations of 0.1 and 1 mg/mL of plant extract were prepared and 0.5 mL of each sample was introduced into test tubes and mixed with 2.5 mL of a 10-fold dilute Folin–Ciocalteu reagent and 2 mL of 7.5% sodium carbonate. The tubes were covered by parafilm and allowed to stand for 30 min at room temperature. The absorbance of the solution was read at 760 nm by the spectrophotometer. All determinations were performed in triplicate. The Folin–Ciocalteu reagent is sensitive to reducing compounds including polyphenols, thereby producing a blue color upon reaction. This blue color is measured spectrophotometrically. Thus, total phenolic content can be determined. The contents were expressed as milligrams of GAE per gram of dried extract [18].

### Phytochemicals compounds

The total phenolic content of the aqueous extract was 197 ± 8 mg GAE per gram of dried extract (mean ± SD). The extract yield was 0.4 g per gram of aerial parts used.

### Preparation of synthetic urine

The composition of synthetic urine was sodium chloride 105.5 mmol/L, sodium phosphate 32.3 mmol/L, sodium citrate 3.21 mmol/L, magnesium sulfate 3.85 mmol/L, sodium sulfate 16.95 mmol/L, potassium chloride 63.7 mmol/L, calcium chloride 4.5 mmol/L, sodium oxalate 0.32 mmol/L, ammonium hydroxide 17.9 mmol/L, and ammonium chloride 0.0028 mmol/L. The synthetic urine was freshly prepared daily and pH adjusted to 6.0 [19].

### Induction of CaOx crystallization

CaOx precipitation was induced by adding 40 μL of 0.1 M sodium oxalate per mL of urine at a temperature of 37 °C and pH 6 in tubes. Urine sample was divided into different aliquots, one of which was used as control (crystallization without plant extract, taraxasterol, and potassium citrate) while in the others, CaOx precipitations were induced in the presence of taraxasterol (2.5, 5, 7.5 and 12.5 μg/mL), *T. officinale* extract (1, 2, 4 and 8 mg/mL), and potassium citrate (100, 150, 200 and 350 mg/mL) which were added to the urine samples before the crystallization process. Lyophilized taraxasterol, *T. officinale* extract, and potassium citrate were resuspended in distilled water, filtered through a 0.22 μm filter, and used at different concentrations.

### Absorbance assay

The inhibitory effects of taraxasterol, extract, and potassium citrate on nucleation of CaOx crystals were determined based on the spectrophotometric assay in different times (2, 4, 6, 8, 10, 20, 30, 40, 50 and 60 min after adding sodium oxalate) at a temperature of 37 °C and pH 6 [20]. The rate of nucleation was determined by comparing the induction time of crystals in the presence of the taraxasterol, extract, and potassium citrate and those of the controls without taraxasterol, plant extract, and potassium citrate. The absorbance of each sample was recorded at 620 nm by the spectrophotometer at different times.

### Inhibition of nucleation

The inhibitory effects of taraxasterol, plant extract, and potassium citrate were calculated as follows:

$$\%inhibition=OD\;\left(control\right)-\frac{OD\;\left(exp\right)}{OD\;\left(control\right)}\times 100$$

Then, the graphs of recorded data were prepared and slope of graphs compared between taraxasterol, plant extract, and potassium citrate.

### Microscopic measurement

After 60 min, the number of crystals in each concentration of taraxasterol, extract, and potassium citrate was measured in five randomly selected fields by the light microscope. Each test was performed in triplicate.

The morphology of crystals was classified as COM (CaC_2_O_4_ monohydrate) and COD (CaC_2_O_4_ dihydrate), then the diameter of COD crystals was measured by the calibrated ocular lens of light microscope. COD crystals were classified according to their diameters into four groups (<5, 5–10, 10–15, and <15 μm). In each specimen, 300 crystals were evaluated by light microscope.

### Statistical analysis

The results are expressed as mean ± SD Statistical analysis and linear regression analysis was performed. The values were analyzed by one-way Analysis of Variance (ANOVA) followed by Tukey’s multiple comparison tests at a significance level of *p <* .05.

## Results

### Absorbance assay

**Figure 1.**
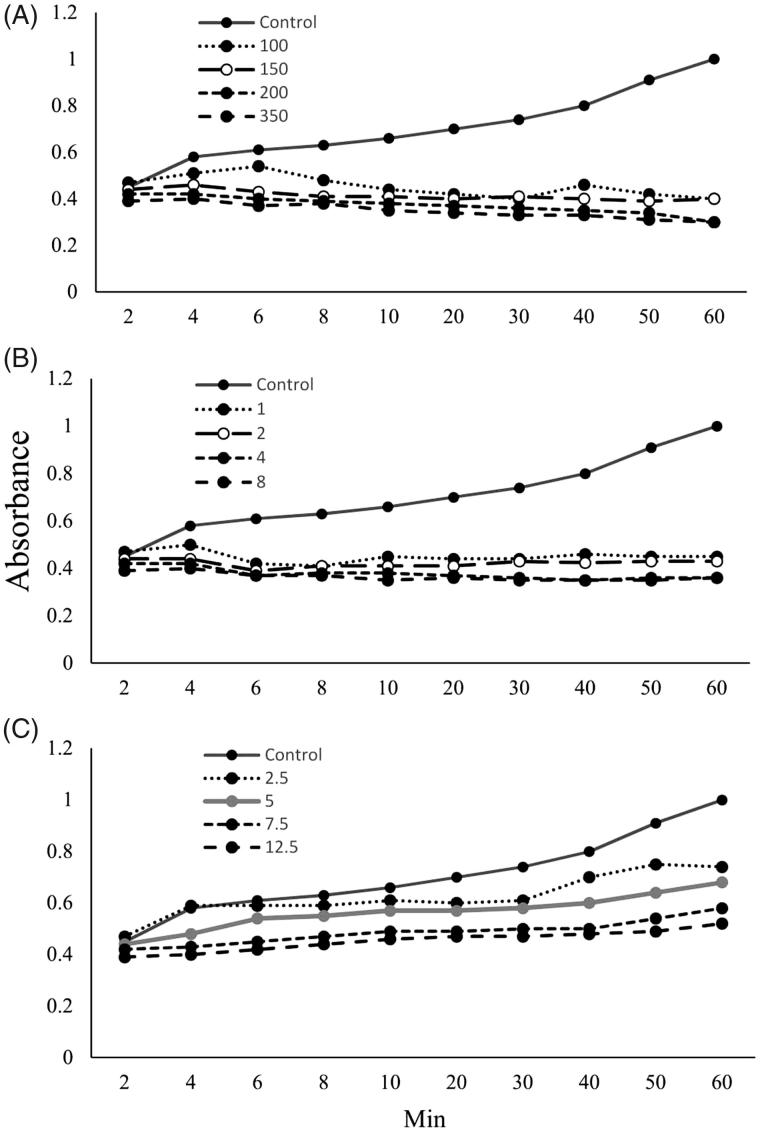
Effects of potassium citrate (100, 150, 200, and 350 mg/mL) (*p* < .01) (A), *T. officinale* extract (1, 2, 4 and 8 mg/mL) (*p* < .001) (B), and taraxasterol (2.5, 5, 7.5 and 12.5 μg/mL) (*p* < .05) (C) on absorbance in synthetic urine specimen.

The results showed taraxasterol (7.5 and 12.5 μg/mL) (*p* < .05), *T. officinale* extract (1, 2, 4 and 8 mg/mL) (*p* < .01), and potassium citrate (100, 150, 200 and 350 mg/mL) (*p* < .001) decreased absorbance in urine specimen (0.45 ± 0.07, 0.36 ± 0.01, and 0.3 ± 0.008, respectively) compared to control (1 ± 0.09). The effects of potassium citrate and extract on absorbance are more potent than taraxasterol, significantly (*p* < .05) (Figure 1).

### Inhibition of nucleation

**Figure 2.**
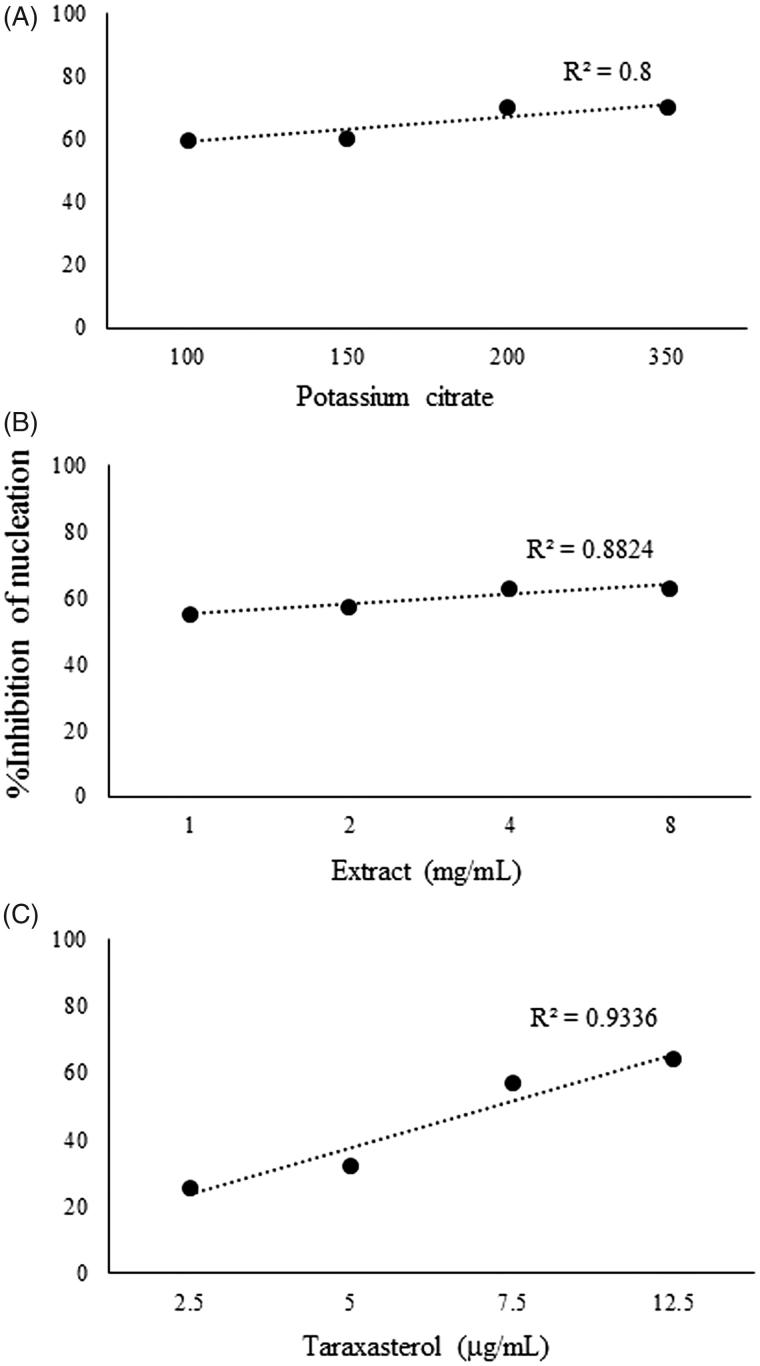
Effects of potassium citrate (100, 150, 200 and 350 mg/mL) (*p* < 0.01) (A), *T. officinale* extract (1, 2, 4 and 8 mg/mL) (B), and taraxasterol (2.5, 5, 7.5 and 12.5 μg/mL) (C) on %inhibition of nucleation of calcium oxalate crystallization in synthetic urine specimen.

The %inhibition of nucleation was increased by taraxasterol, extract of *T. officinale*, and potassium citrate in the range of 26–64, 55–63 and 60–70%, respectively. There were dose-dependent increases in %inhibition of nucleation by taraxasterol (*r*^2 ^= 0.8), the extract of *T. officinale* (*r*^2^ = 0.8824), and potassium citrate (*r*^2 ^= 0.9336). The effects of potassium citrate and extract on %inhibition of nucleation are more potent than taraxasterol, significantly (*p* < .05) (Figure 2).

### Microscopic analysis

**Figure 3.**
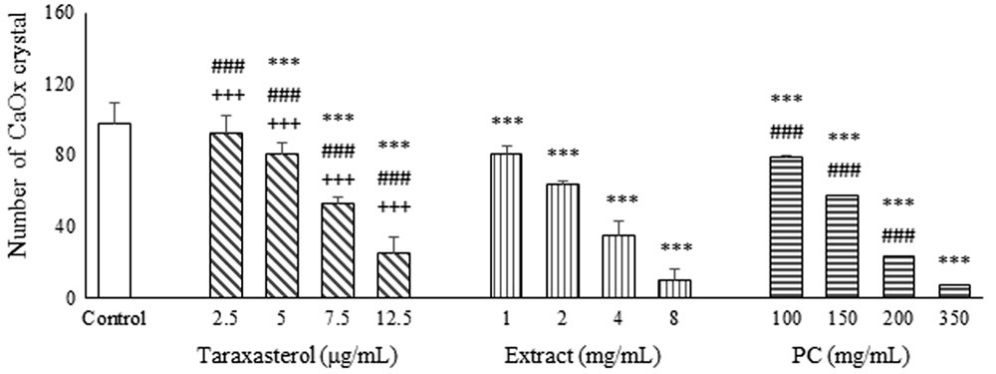
Effects of taraxasterol (2.5, 5, 7.5 and 12.5 μg/mL), *Taraxacum officinale* extract (1, 2, 4 and 8 mg/mL) and potassium citrate (100, 150, 200 and 350 mg/mL) on number of CaOx crystals. *p < .05, **p < .01, and ***p < .001 different from control.

**Figure 4.**
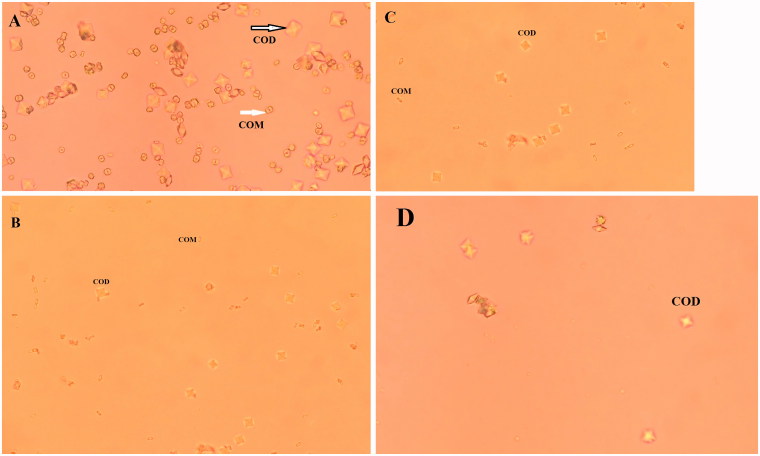
Light microscopic photographs of crystal formation in control group (A), presence of taraxasterol (B), *Taraxacum officinale* extract (C), and potassium citrate (D) (×400). COD: CaOx dihydrate (bipyramidal-shaped) and COM: CaOx monohydrate (oval-shaped).

The result showed taraxasterol (7.5 and 12.5 μg/mL), extract (4 and 8 mg/mL), and potassium citrate (150, 200 and 350 mg/mL) caused significant decreases in the number of CaOx crystals compared to control group in a dose-dependent manner (Figures 3 and 4). The effects of potassium citrate and extract on the number of CaOx crystals are more potent than taraxasterol, significantly (*p* < .001).

Also, the light microscopic results showed that addition of sodium oxalate (0.1 M) to synthetic urine samples produced COM (oval-shaped) and COD (bipyramidal-shaped) crystals (Figure 4). It was found that taraxasterol, *T. officinale* extract, and potassium citrate decreased the number of COM and increased COD crystals in experimental urine samples compared to control sample in a dose-dependent manner. Also, the size of COD crystals was decreased in the presence of taraxasterol, extract, and potassium citrate, significantly. Effects of *T. officinale* and potassium citrate on the number of COM and COD crystals are more potent than taraxasterol, significantly (*p* < .001) (Table 1).

**Table 1.** Effects of potassium citrate, aqueous extract of *T. officinale* and taraxasterol on the number and morphology of CaOx crystals (COM and COD) and the diameter of COD crystals.

		COD			
	COM	<5 μm	5–10 μm	10–15 μm	>15 μm
Control	195 ± 45	11 ± 4	53 ± 7	25 ± 9	11 ± 2
Potassium citrate (mg/mL)					
100	180 ± 73	46 ± 4***	54 ± 12	13 ± 8***	7 ± 2***
150	156 ± 66	64 ± 10***	51 ± 16	23 ± 5+++	6 ± 1***
200	109 ± 38***	125 ± 13***	47 ± 8	17 ± 4+,#	2 ± 0.5***
350	45 ± 21***	197 ± 36***	40 ± 5	15 ± 3**	3 ± 1***
Extract (mg/mL)					
1	212 ± 43	23 ± 8+++	44 ± 11*	11 ± 5***	10 ± 3
2	190 ± 32	41 ± 9***,+++	50 ± 13	12 ± 4***	7 ± 2***
4	175 ± 67	68 ± 19***,+++	45 ± 15	9 ± 5***	3 ± 1***
8	118 ± 49***,++	128 ± 10***,+++	42 ± 9	8 ± 2***	4 ± 2***
Taraxasterol (μg/mL)					
2.5	247 ± 31	18 ± 3###,+++	11 ± 5***,+++	12 ± 2***	12 ± 4
5	203 ± 48	29 ± 10*,###,+++	31 ± 9***	24 ± 4+	13 ± 5
7.5	147 ± 61	48 ± 15***,###,+++	42 ± 19	48 ± 13***,+++	15 ± 5
12.5	125 ± 58**,+++	59 ± 25***,###,+++	53 ± 18	52 ± 16***,+++	11 ± 6

*p < .05, **p < .01, ***p < .001 different from control.

+*p* < .05, ++*p* < .01, +++*p* < .001 different from potassium citrate (350 mg/mL).

#*p* < .05, ###*p* < .001 different from extract (8 mg/mL).

## Discussion

Supersaturation is determined as a solution which contains more of dissolved materials that could be dissolved by the solvent in normal conditions, which results in the formation of the crystals and subsequently production of nuclei for attachment of the newly crystals [7]. Crystal formation in urinary tubules begins with the supersaturation of kidney stone materials. It indicates that the concentration of stone materials in urine is higher than the thermodynamic solubility of those materials. The force for crystallization is a reduction in the potential energy of the atoms or molecules when they bind together. Since the lumen space of renal tubules is not enough for crystal nuclei production, so they cannot become large and pass through tubules and enter to the renal pelvis. In the renal pelvis, they can aggregate into large clumps and finally these aggregate of crystals retained in the urinary tract [21]. Thus, if one of the steps of crystal formation in urine can be inhibited, urolithiasis can be prevented.

The reports indicated that the organic materials with inhibitory effects on kidney stone can adsorb to the surface of the crystals and then cover them. So, they can inhibit one of the steps of urine crystallization, such as crystal nucleation, growth, and aggregation [8]. Glycosaminoglycans [22], citrate, magnesium, and orthophosphate [23], are known as potent inhibitors of CaOx crystallization, but they have high molecular weights and their clinical usages have been limited, so they cannot be increased in urine.

Traditional medicine can be exhibited by increasing the urine volume and pH or balancing the processes of inhibition and promotion of nucleation, aggregation, and growth of crystals in urine. Herbal medicines contain various constituents, so they can exert beneficial effects by relieving the binding mucin of calculi or lithotriptic activity, regulation of oxalate metabolism, or regulation of the crystalloid-colloid imbalance, and thereby preventing the recurrence of urinary calculi [24].

The present results showed that absorbance of urine samples was decreased in presence of taraxasterol (*p* < .05), plant extract (*p* < .01), and potassium citrate (*p* < .001). Also, taraxasterol, extract, and potassium citrate inhibited nucleation of *in vitro* CaOx crystallization, significantly.

The results showed that taraxasterol, plant extract, and potassium citrate decreased the number of CaOx crystals, the number of COM crystals, increased the number of COD crystals and also decreased the size of COD crystals compared to control sample, significantly (*p* < .001). In all experiments, the effects of potassium citrate and plant extract are more potent than taraxasterol.

Taraxasterol, *T. officinale* extract, and potassium citrate may have special materials which can exert direct or indirect actions on the number and morphology of CaOx crystals.

There are some published reports similar to this study about inhibition of nucleation of CaC_2_O_4_ crystals by the plants including the extract of *Terminalia arjuna* (Roxb.) *Wight & Arn.* (Combretaceae) bark [25], *Dolichos biflorus* L. (Fabaceae) seeds [26], *Phyllanthus niruri* L. (Phyllanthaceae) [27], *Beta vulgaris* L. (Amaranthaceae) leaf and root [28], *Alismatis rhizoma* L. (Alismataceae) [4], *Bergenia ligulata* (Wall.) Engl. (Saxifragaceae) rhizome [29], *Tribulus terrestris* L. (Zygophyllaceae) [30].

Wesson et al. showed the presence of *Phyllanthus niruri* extract induced alterations in CaOx crystal morphology, favoring the formation of the COD form, which is less likely to bind to renal cells [31].

It is shown that COM crystals have higher adhesion affinity to renal epithelial cells than COD crystals [11]. It may constitute the form of higher potential risk for stone formation. Therefore, the appearance of more COD than COM particles in experimental samples may be a good result.

Urinary calculi are composed of the organic matrix and different amounts of the crystalloids which located onto or within the matrix. The natural products inhibit the initial precipitation of calcium and phosphate ions in the form of a mineral phase bound to the organic matrix and then prevent the growth of the crystal [32]. Thus, the extract of plants might contain compounds that could stimulate the demineralization of the matrix-bound mineral phase. The anti-urolithiatic property of the plants was probably due to their phytoconstituents.

Furthermore, it is reported that *Achyranthes indica* L. (Amaranthaceae) hydroalcoholic extract inhibited the CaOx crystal nucleation and aggregation. The ability of extract to reduce the nucleation increases the metastable limit of oxalate in urine and prevents the precipitation of the CaOx crystal [33].

The major phytoconstituents of *T. officinale* extract are tocopherols, phenols, flavonoids, saponins and sterols [34], major sesquiterpene lactones including taraxasterol, and oligoelements (calcium, sodium, magnesium and potassium) [35]. Flavonoids have been reported to prevent CaOx crystallization in urine and animal models [36] and inhibit CaOx crystal deposition [37]. However, the rule of elements of *T. officinale* including magnesium and potassium cannot be ruled out. Also, saponins are also known to possess anti-crystallization properties *via* disaggregation of the suspension of mucoproteins and promoters of crystallization [38]. Therefore, it can be hypothesized that the effect of *T. officinale* extract could be due to the presence its constituents including taraxasterol.

## Conclusion

It is concluded the aqueous extract of *T. officinale* and taraxasterol were able to inhibit CaC_2_O_4_ crystallization *in vitro* and the potential of taraxasterol was less than *T. officinale* and potassium citrate which could be because of other active components of extract with the synergistic effect. The kidney stone formation is not only a crystallization phenomenon, but cellular processes in the renal tissue especially at the papillae play even more decisive roles.
